# Unpaired MR-CT brain dataset for unsupervised image translation

**DOI:** 10.1016/j.dib.2022.108109

**Published:** 2022-03-30

**Authors:** Omar S. Al-Kadi, Israa Almallahi, Alaa Abu-Srhan, A.M. Mohammad Abushariah, Waleed Mahafza

**Affiliations:** a King Abdullah II School of Information Technology, The University of Jordan, Amman 11942, Jordan; b Department of Basic Science, The Hashemite University, Zarqa, Jordan; c Department of Diagnostic Radiology, Jordan University Hospital, Amman 11942, Jordan

**Keywords:** MR and CT image dataset, Image translation, Unsupervised learning, Brain tumors

## Abstract

The data presented in this article deals with the problem of brain tumor image translation across different modalities. The provided dataset represents unpaired brain magnetic resonance (MR) and computed tomography (CT) image data volumes of 20 patients. This includes 179 two-dimensional (2D) axial MR and CT images. The MR cases are acquired using Siemens Verio scanner, while the CT images with a Siemens Somatom scanner. The MR and CT tumor volumes were collected, diagnosed and annotated by experienced radiologists specialized in oncology and radiotherapy. The collected image volumes can be useful for researchers working in the field of artificial intelligence (AI) applications for brain tumor detection, classification and segmentation in MR and CT modalities. The provided tumor masks per each tumor volume can assist data scientists with limited background in cancer imaging. Moreover, clinical interpretation is given per each tumor volume, which can assist in deep learning model training with multiple source data (non-imaging or textual data) as well. The provided dataset can facilitate for annotation-efficient lesion segmentation using bidirectional MR-CT cross-modality image translation.

## Specifications Table

**Table:** 

Subject area	Medical Imaging Analysis
Specific subject area	Brain MR and CT scans
Type of data	MR and CT image volumes (along with lesion description in Table 1 and segmentation masks in Table 2)
How data were acquired	Data is collected using Siemens Verio scanner for the MR images, and Siemens Somatom scanner for the CT images.
Data format	Raw DICOM image files
Parameters for data collection	MR: 3 Fat sat pulses (FS), 2500-4000 TR, 20-30 TE, and 90/180 flip angle CT: topogram acquisition protocol, 2.46 mGY.cm dose length, 113-327 mAs tube current, 130 KV voltage, 64 dual source, one projection, and slice thickness of 7.0 mm
Description of data collection	MR and CT image volumes were acquired at the Department of Diagnostic Radiology – Jordan University Hospital (JUH) – between the period of April 2016 and December 2019. The MR and CT tumor volumes were collected, diagnosed and annotated by experienced radiologists specialized in oncology and radiotherapy.
Data source location	Jordan University Hospital, P.O. Box 1304, 11942, Amman, Jordan
Data accessibility	Repository name: Mendeley Data; Data identification number: 10.17632/z4wc364g79.1; Direct URL to data: https://dx.doi.org/10.17632/z4wc364g79.1
Related research article	A. Abu-Srhan, I. Almallahi, M. Abushariah, W. Mahafza, O. Al-Kadi, Paired-Unpaired Unsupervised Attention Guided GAN with Transfer Learning for Bidirectional Brain MR-CT Synthesis, Computers in Biology and Medicine, 136 (2021). https://doi.org/10.1016/j.compbiomed.2021.104763

## Value of the Data

• The provided annotated MR and CT image volumes can be useful for categorizing and labeling of human brain lesions for AI clinical applications; particularly, supporting automated medical image analysis for early brain tumor detection.
• Labeling medical images is generally challenging, time-consuming, tedious, and requires labor-intensive participation of radiologists; the dataset can assists in dealing with medical data scarcity for training deep learning models, specifically for MR and CT images.
• The dataset comes along with detailed clinical description for each of the MR and CT lesion cases (see Table 1), which can provide better understanding of lesion physical characteristics.

**Table 1:** Lesion description.

Patient	Age	F/M	CT Lesion Description	MR Lesion Description
1	35	M	Large well-defined hypodensity occupying most of the right parietal and temporal lobes, causing mass-effect on adjacent structures inform of diffuse sulci effacement of the right brain hemisphere, compression of ipsilateral lateral ventricle and causing mid line shift about 5 mm to the left.	Abnormal signal intensity involving the grey and white matter in the lateral aspect of the right temporal lobe (at and around the right middle temporal gyrus), associated with a gross expansion and mass-effect with no post contrast enhancement consistent with neoplastic process.
2	60	M	A large supratentorial heterogenous irregular left basal ganglia abnormality with haemorrhagic component surrounded by cytotoxic oedema exerting mass-effect with complete obliteration of anterior horn of left lateral ventricle as well as third ventricle.	Also demonstrates CT lesion with multifocal acute haemorrhagic components, Histopathologic results shows reactive gliosis.
3	45	F	Three extra-axial well-defined hyperdense mass lesions seen on the right frontal parasagittal, left frontal and right parietal regions, the right frontal lesion appeared to be surrounded by minimal vasogenic oedema with mild mass-effect upon the right frontal horn of lateral ventricle	avid enhancement of these 3 lesions, Histopathologic Result shows meningioma.
4	45	F	Left temporoparietal ill-defined heterogenous tumour with hyperdense component, this is surrounding by vasogenic oedema causing midline shift to the right side of about 9.2 mm as well as dilatation comperssion upon the left lateral ventricle and 3rd ventricle.	The known left temporoparietal tumour with foci of enhancement, Histopathologic Result shows grade III Astrocytoma.
5	28	M	Large heterogenous soft tissue lesion with cystic component and foci of calcification seen occupying the fourth ventricle with dilatation of lateral, third ventricles and upper part of forth ventricle, in addition to periventricular hypodensity suggestive of active hydrocephalus.	The known fourth ventricle lesion with solid, cystic and calcific component and heterogeneous postcontrast enhancement, it also extends along the Luschka foramina especially on the left side, No Histopathologic Result was avaliable but radiologicaly features.may suggest medulloblastoma vs ependymoma.
6	70	M	Irregular hypodense White matter lesion seen at the right suprainsular frontal lobe with surrounding vasogenic oedema, this measured 2.5 x 3 cm.	Multiple heterogeneous lobulated, peripherally ring enhancing lesion, Histopathologic Result result showed infiltrating astrocytoma grade grade IV.
7	38	F	Extra-axial cystic lesion attached to skull base at right cavernous sinus in close relation to the right Meckel’s cave and possibly arising from it, although the Meckel’s cave appeared compressed, in addition there is marked mass-effect in the brain with compression of brainstem.	Cystic with peripheral soft tissue component more evident medially, with peripheral enhancement, Histopathologic result showed Schwannoma.
8	63	F	Extensive vasogenic oedema involving the right temporofrontoparietal region, with suspicious underlying intra-axial heterogenous mass with its epicentre at the right temporal lobe, in addition there is also compression of the ipsilateral cerebral peduncle and mild deviation of the midbrain to the left.	Irregular heterogenous predominantly peripherally enhancing soft tissue component. Histopathologic Result result showed Grade IV infiltrative astrocytoma.
9	59	F	A well-defined hypodense lesion seen at the left CP angle with dura attachment to left side of tentorium, this mass exerting mass-effect on the brainstem,compressing basal cisterns shifting them on the right side.	Intense homogenous enhancement, No Histopathologic Result was avaliable but radiologicaly features mostly represent Meningioma.
10	63	M	Large intra-axial lesion seen in the right frontal lobe anteriorly, with cystic and gross calcific portions, surrounded by prominent vasogenic oedema that caused compression of the ipsilateral lateral ventricle and shift of the midline to the left side of about 6 mm.	Heterogenous mainly cystic lesion with peripheral rim enhancement, histopathologic results shows Glioblastoma multiforme.
11	30	F	Right temporal well-defined hypyerdense lesion with marked surrounding vasogenic oedema compressing the ipsilateral lateral ventricle with midline shift to the left side, in addition there is evidence of craniotomy sutures at the same level.	Intense enhancement of the lesion with an adjacent overlying leptomeningeal enhancement, Histopathologic results showed central nervous system embryonal cell grade IV.
12	56	M	A suprasellar heterogenous mainly hypodense lesion with peripheral calcifications and exerting mass-effect on the surrounding measured 2.5 x 2.3 cm.	Peripheral diffusion restriction and faint central enhancement along with peripheral rim enhancement, Histopathologic results showed epidermoid cyst.
13	35	F	An ill-defined isodense lesion with foci of calcification seen at the left temporal lobe with surrounding vasogenic oedema with evidence of adjacent craniectomy.	Irregular meningeal enhancement at the left temporal lesion with surrounding vasogenic oedema. Histopathologic results showed Meningioma WHO grade 1.
14	54	M	Lobulated hyperdense lesion seen in the right parasagittal frontal lesion with meningeal dural pain measuring by MR slightly compressing adjacent right frontal lobe with minimal vasogenic oedema.	Avid contrast enhancement for this lesion.Histopathologic results result shows Atypical meningioma WHO grade II.
15	50	F	A well-defined hyperdense,extra-axial dural based lesion seen at the right parietal lobe measuring 3 x 5 cm, causing compression of the ipsilateral lateral ventricle.	Avid contrast enhancement for the right parietal lesion, Histopathology showed Meningioma WHO grade 1.
16	30	F	Multiple bilateral supratentorial intraparenchymal hyperdense space-occupying lesions noted with surrounding vasogenic oedema, consistent with the known CNS lymphoma, one of the lesions seen in the right frontal region developed haemorrhagic component and caused Midline shift to the right side about 8 mm.	An irregular heterogenous predominantly peripherally enhancing soft tissue component and restricted diffusion. Histopathology showed B cell lymphoma.
17	45	M	Lobulated extra-axial dural based lesion the left frontal convexity with associated vasogenic oedema,it measured 5 x 4.5 x 3.3 cm.	Homogenous postcontrast enhancement. Histopathologic results showed Meningioma WHO grade II.
18	59	M	A heterogenous round soft tissue mass lesion at parasagittal area of right frontal lobe with extensive surrounding vasogenic oedema, this showed areas of fluid density. It crossed the midline and caused midline shift to the left side of about 12 mm.	A large rounded lesion seen at the right frontal lobe which appeared. This lesion crosses the midline and causing midline shift to the left side of about 11 On diffusion weighted images. Histopathology showed glioblastoma multiforme (WHO IV).
19	47	F	A large olfactory groove extra-axial iso to hyperdense mass lesion with foci of calcification that measures about 7x6x6 cm,with surrounding vasogenic oedema in the anterior frontal lobes bilaterally. It is associated with significant mass-effect with posterior displacement of the midline structures particularly corpus callosum and optic chiasm.	A large olfactory groove mass and surrounding vasogenic oedema in the anterior frontal lobe bilaterally. Associated with significant mass-effect, after contrast injection avid enhancement is noted. Histopathology pathology olfactory meningioma (WHO II).
20	34	F	Intraparenchymal hyper dense lesion seen occupying the left putman with surrounding marked vasogenic edema,in keeping with interaparanchymal hemorrhage.	Heterogenous putman Lesion with high signal intensity corropending to foci of hemorrhage.

**Table 2:** Segmentation tumor mask for MR and CT images.
• The collected MR and CT volumes, along with provided tumor masks (see Table 2), can be used by data scientist to generate realistic MR images from CT images and vice versa for learning reconstruction models [1].

## 1 Data Description

The MR-CT brain image volumes were acquired by the Diagnostic Radiology Department of the Jordan University Hospital (JUH). The dataset was acquired between the period of April 2016 and December 2019. The dataset consists of brain CT and MR image volumes scanned for radiotherapy treatment planning for brain tumors. The dataset contains T2-MR and CT images for 20 patients aged between 26-71 years with mean-std equal to 47-14.07. Lesion masks were manually delineated by two expert radiologists using a software tool developed in python. Our tool allows for scrolling through all image slices and adjustment of window level settings before selecting the segmentation area. More information can be found in [1]. The MR and CT volume scans are described as follows:

## 2 Experimental Design, Materials and Methods

### 2.1 MR and CT scanner configuration

The MR images of each patient were acquired with a 5.00 mm T Siemens Verio 3T using a T2-weighted without contrast agent, 3 Fat sat pulses (FS), 2500-4000 TR, 20-30 TE, and 90/180 flip angle. The CT images were acquired with Siemens Somatom scanner with 2.46 mGY.cm dose length, 130 KV voltage, 113-327 mAs tube current, topogram acquisition protocol, 64 dual source, one projection, and slice thickness of 7.0 mm. Smooth and sharp filters have been applied to the CT images. The MR scans have a resolution of 0.7×0.6×5 mm${}^{3}$, while the CT scans have a resolution of 0.6×0.6×7 mm${}^{3}$.

### 2.2 Data collection

The dataset consists of 2D image slices extracted using the RadiAnt DICOM viewer software. The extracted images are transformed to DICOM image data format with a resolution of 256×256 pixels. There are a total of 179 2D axial image slices referring to 20 patient volumes (90 MR and 89 CT 2D axial image slices). The dataset contains MR and CT brain tumour images with corresponding segmentation masks [2].

### 2.3 Shared files and directory structure

The MR-CT dataset is organized in two folders (MR and CT). Each folder consists of two sub folders (images and corresponding masks). The DICOM images in each sub folder are formatted according to the following naming convention: CASE NUMBER | IMAGE TYPE (CT OR MR) | VOLUME SLICE OR SEGMENTATION MASK NUMBER| FILE EXTENSION | where S indicates image slice and M is the associated segmentation mask.

## Ethics Statement

The Jordan University Hospital MR-CT Brain Dataset has been collected after receiving Institutional Review Board approval (IRB no. 16/161/2020) and the consent of patients. All procedures has been carried out in accordance with The Code of Ethics of the World Medical Association (Declaration of Helsinki).

## CRediT authorship contribution statement

**Omar S. Al-Kadi:** Conceptualization, Methodology, Supervision, Writing – review & editing. **Israa Almallahi:** Data curation, Formal analysis, Writing – original draft. **Alaa Abu-Srhan:** Investigation, Software, Data curation, Writing – review & editing. **A.M. Mohammad Abushariah:** Supervision. **Waleed Mahafza:** Conceptualization, Validation.

## Declaration of Competing Interest

The authors declare that they have no known competing financial interests or personal relationships which have, or could be perceived to have, influenced the work reported in this article.

## Data Availability

Unpaired MR-CT Brain Dataset for Unsupervised Image Translation (Original data) (Mendeley Data).
